# Traditional Islamic spiritual meditative practices: powerful psychotherapies for mental wellbeing

**DOI:** 10.3389/fpsyg.2025.1538865

**Published:** 2025-05-16

**Authors:** Farah R. Zahir, M. Walid Qoronfleh

**Affiliations:** ^1^Irfa’a Foundation, Burlington, ON, Canada; ^2^University of British Columbia, Vancouver, BC, Canada; ^3^Healthcare Research & Policy Division, Q3 Research Institute (QRI), Ypsilanti, MI, United States

**Keywords:** spirituality, Islam, mental health, wellness, meditation, dhikr, Quran, Unani Tibb

## Abstract

The new millennium is witnessing a remarkable shift within the scientific community, to be increasingly understanding and accepting the impact of meditative, spiritual, and even religious practices, on health and wellbeing. This has been precipitated ironically, by cutting edge scientific studies. Rigorous empirical research in epigenomics and neuroscience is proving that religious and spiritual experiences impact psychology and physiology. Therapeutic and prophylactic benefits from spiritual meditative practices (SMPs) are now proven for a variety of chronic conditions, notably in mental health wellness. While all forms of SMPs are currently being investigated, those from the Islamic tradition are lagging behind interrogation of similar SMPs from other traditions, such as mindfulness. Traditional Islamic SMPs have remained largely hidden or misunderstood, and as yet poorly translated into the modern context. In this paper we situate Islamic SMPs in the context of how they impact mental health and wellbeing, explaining their breadth and depth. We highlight the efficacy of dhikr and Quran recitation therapies as treatments for addiction and anxiety, noting historic evidence, and discuss how they may be integrated into modern mental health treatments. A millennium and a half of historical data proves their efficacy as psychotherapy. Precedent for the use of Islamic SMPs to treat mental illness for all people regardless of faith exists, highlighting their potential for wide implementation today. Of the Islamic SMPs that can most easily be accessed by modern people, listening to Quranic recitation and forms of dhikr therapy are notable for their ease of administration and strikingly uniform positive results. More rigorous empirical studies are called for to better translate Islamic SMPs into modern complimentary and alternative medicine, as they hold great promise as universally adoptable cogent modern psychotherapies.

## Introduction

Religion and spirituality are integral aspects of the human experience, deeply influencing mental health and overall well-being. Similarly, science, in its broadest sense, has always been central to human advancement. Despite this, religion and science are commonly considered to be opposing philosophies, especially with the rise of secularism originating in renaissance Europe and post-modern Westernism. In contrast, the Islamic world-view has always considered religion and science to be complementary. Islam encourages the study of nature, reflection on creation, and the pursuit of knowledge, illustrating how religion and science can thrive together. Quranic injunctions encouraging human beings ‘to think’, ‘to reason’, ‘to contemplate’ are numerous, resulting in critical thinking accepted as normative (Malik, 2017; Ramli et al., 2018; Junoh et al., 2021). Therefore, Islamic civilization was particularly prescient in its’ pursuit of studies into the neurosciences, such as cognition, psychiatry and psychology. This is especially evident during the historical period known as the Golden Age of Islam (Mohamed, 2008),

On the other hand, the sometimes stark demarcation between religion and science in Western civilization, was particularly notable during the 20th century. However, in recent years a shift toward greater acceptance of religion, particularly of spirituality, within the scientific community is becoming apparent as exemplified by the emerging field of research termed ‘neurotheology’ (Dixon and Wilcox, 2016; Al-Nuaimi et al., 2020; Bragazzi et al., 2018). Furthermore, the publication of the World Health Organization’s (WHO) traditional medicine strategy document at the dawn of the new Millenium (World Health Organization, 2002) underscores the resurgence of complimentary and alternative medicines (CAM), including their strong integration of spiritual practices, as legitimate therapeutic practices. The WHO recognized three main traditional medicine systems as legitimate CAMs; ‘Unani Tibb’ (UT), centered in the Muslim world as their traditional medical system, Traditional Chinese Medicine (TCM) and Ayurvedic Medicine (AM). While TCM and AM are familiar to most audiences, UT with its strong emphasis on spiritual and meditative practices as part of the healing and wellness regimen, is less well known.

In this paper we explore the psychological and physiological impacts of spirituality on human health particularly from the lens of UT, and the Islamic religious tradition. We first highlight spiritual meditative practices’ (SMPs) impact on mental wellness. We then introduce and comment on the extent and scope of Islamic religious and spiritual practices. Contextualizing them as potential psychotherapies capable to build psychological resilience and effect psychiatric treatment. While still largely unfamiliar to western science, they are cogent tools for mental wellbeing applicable to all peoples (regardless of faith) and therefore must be extensively explored.

### Spiritual meditative practices (SMPs) as a means to enhance health and wellness

The first two decades of the new millennium have witnessed a burgeoning interest in ancient and traditional practices that enhance human health and wellbeing. A steadily increasing body of literature is extant demonstrating effective positive psychological, and even biological impacts for spiritual, meditative or contemplative practices (Glicksohn et al., 2020; Mitha, 2019; Mohamad et al., 2020; Schulz and Waldinger, 2023; Zahir, 2022). We will call these spiritual meditative practices (SMPs) for the purposes of this article. Mindfulness (Kabat-Zinn, 1990, 2019) based CAM therapies such as Mindfulness Based Stress Reduction (MBSR), and Mindfulness Based Cognitive Therapy (MBCT) are some of the most recognizable among them (Alsubaie et al., 2017; Pedro et al., 2021). While currently practiced secularly, they are based upon Buddhist spiritual exercises designed to advance consciousness while also impacting biological and psychological processes (Ling, 2021).

Concomitantly, the wellness sphere has seen enormous growth in the past two decades, with it now being accepted as essential to mental and physical wellbeing. Wellness bears especial importance as a preventative measure against the growing incidence of mental illness witnessed by affluent societies (Christensen et al., 2020; Scott et al., 2021). Yoga is a form of mind–body wellness SMP that originated in the AM tradition, that is now almost universally practiced. While its current global implementation is largely secular, in its original and true form it is a powerful spiritual advancement-training from the ancient Vedic-Hindu religious tradition. Similarly, qigong and tai-chi are two other prominent mind–body SMPs originating from the Daoist/Buddhist spiritual traditions that are now undertaken by diverse populations as a form of secular wellness practice.

### Current research evidences that SMPs are able to affect physiology, notably via epigenomic modulation

Numerous studies are demonstrating that the above SMPs are able to affect beneficial biological and psychological changes in the human body (Buric et al., 2017; Klimecki et al., 2019; Glicksohn et al., 2020). Notably, they are able to impact metabolic pathways prominent in chronic disease (Zahir, 2022). A recent systematic review of gene expression studies for meditative practices interrogated 18 rigorous studies that met their inclusion criteria, spanning SMPs that included MBSR, transcendental meditation, mindfulness, yoga, tai chi and qigong. All reported positive gene expression changes pertaining to metabolic pathway biomarkers, with specifically, a downregulation of the NF-κB stress response pathway commonly identified (Buric et al., 2017). The NF-κB pathway is notable for being upregulated during stressful situations, and if turned on for extended periods of time can lead to occurrence of several chronic diseases such as anxiety, obesity, type 2 diabetes, depression and even certain cancers (Hotamisligil and Davis, 2016; Zahir, 2022). Corroborating findings from numerous studies of SMPs conclude that they are able to reduce anxiety and stress, a few are cited here (Babamohamadi et al., 2015; Ghiasi and Keramat, 2018; Sulistyawati and Setiyarini, 2019; Spinhoven et al., 2022).

Notably, a key mechanism by which SMPs are able to affect pathophysiology of the human body is via causing changes in the epigenomic profile and programming (Kaliman, 2019; Zahir, 2022). Epigenetic processes are known to be a major method by which the environment alters gene functioning (Tammen et al., 2013). This alteration can be dynamic as well as static, and is recognized to be a highly sophisticated means of modulating the genome according to environmental stimuli (Perera and Herbstman, 2011; Tammen et al., 2013). It has been shown that SMPs are able to counter damaging effects from the environment that often lead to chronic disease. For example, a narrative review of studies probing the health benefits of yoga (a mind–body-meditation that has close resemblance to the Islamic mandatory worship mind–body-meditation ritual named salat (Sayeed and Prakash, 2013) discussed below) showcases 11 randomized control trials of yoga versus a variety of other health interventions for several chronic conditions. Among key findings were positively altered epigenetic markers for genes involved in inflammation, stress response pathways including NF-κB, DNA damage repair pathways, and telomere length related cellular ageing (Giridharan, 2023). Participants included those with type 2 diabetes, individuals with chronic lung conditions, glaucoma patients, and individuals in psychological distress (Giridharan, 2023).

In the sphere of mental illnesses, clear empirical evidence exists proving that SMPs produce therapeutic benefits (Rubia, 2009; Shen et al., 2020; Magan and Yadav, 2022). SMPs from the Islamic tradition are frequently reported to cause mental wellness impacts (detailed below) with suggested pathophysiology centered on epigenomic process alteration (Zahir, 2022).

In contrast to psychotherapies originating from the Buddhist/Hindu/Vedic axes, those from the Islamic tradition are yet poorly translated into modern secular awareness (Zahir, 2022). Notably, UT features Islamic SMPs as an integral healing therapy, more so than found within TCM or AM. As Islamic SMPs, especially with respect to how they relate to non-Muslims, are not well known, they are briefly overviewed next.

### Islamic SMPs – traditionally evidenced health benefits

Islamic SMPs comprise a vast and varied compendium that include both mandatory mind–body SMP worship rituals as well as a plethora of voluntary SMPs. They may be described as hidden and misunderstood in that many of them are not commonly known, even to modern day Muslims. Most Muslims are familiar with ritualized meditative worship acts that are mandatorily a part of their faith. These are the ‘five pillars of Islam’; (a) to make an intentional declaration of the Oneness of the Divine and the prophethood of Muhammed as the final messenger of the Divine, (b) to make a mind–body-meditation worship ritual known as ‘salat’ five times a day according to periods determined by the sun’s movement (this salat also involves an important ritualized form of washing called ‘wudu’ that is mandatory), (c) to fast from all food, drink, and sexual activity from sunrise to sunset during the Islamic month of Ramadan, (d) to offer 2.5% of one’s wealth beyond a certain threshold as alms annually, and, (e)to make the Hajj pilgrimage at least once in one’s lifetime if able (Hitchcock, 2005; Raheema and Omar, 2018; Zaw et al., 2016).

In addition to the above, there are other countless forms of voluntary SMPs that may be collectively termed ‘dhikr’ (Nurbakhsh, 1978; Chishti, 1985; Ridgeon, 2015; Saniotis, 2018; Mitha, 2019; Sulistyawati and Setiyarini, 2019). These are often esoteric, and almost always traditional. The Arabic word ‘dhikr’, may be translated as ‘remembrance’. Used in the spiritual context it denotes all forms of spiritual practices intended to bring about a state of harmony with the Divine. To contextualize the word, it may be considered the equivalent of ‘meditation’ in the Buddhist tradition, or to secular practices for ‘being present’ or ‘mindful’. Thus, one understands that as much as there are meditation types, or mindfulness mores, there exist forms of dhikr. More prominent among them are; (a) reciting the Quran in Arabic, or listening to it being chanted (Ghiasi and Keramat, 2018; Yadak et al., 2019), (b) intoning/chanting specific religious litanies repeatedly. This may be done in a number of ways, some including bodily movement and musical accompaniment while others are still and silent (Chishti, 1985; Mitha, 2019), and (c) forms of breathing undertaken in conjunction with the recitation of ‘names’ of the Divine (Nafisa et al., 2017). Correctly speaking, all forms of worship in the Islamic tradition are indeed dhikr, even the aforementioned mandatory ‘five pillars’. However, dhikr is commonly used to refer to non-mandatory SMPs such as those outlined in this paragraph.

Importantly, all forms of dhikr are persuasive vehicles to elevate the consciousness to higher levels of closeness with the Divine (Ahmad, 2012; Bragazzi et al., 2018), or in other words, are potent spiritual effectors. Significantly, all forms of dhikr have been accepted for centuries to have powerful healing properties by generations of Muslims, and feature prominently in UT. They are known to not only heal mental and bodily illnesses but also to exert strong prophylaxis, especially against the onset of mental disorders.

Muslim traditional physicians who practice UT are knowns as ‘hakims’. Hakims have been prescribing forms of dhikr for centuries in their practice as a form of medicine (Sheehan and Hussain, 2002; Hoosen, 2017; Itrat, 2020; Khan et al., 2022). Remarkably, dhikr was prescribed not only for Muslim, but also for non-Muslim patients, demonstrating that adherence to the Islamic faith is not a prerequisite for the dhikr to work. Indeed, the UT medical system was extant all across the Muslim world prior to the colonial conquests that vastly decimated the rich intellectual, academic, and cultural centers of the Muslim world from West Africa to East Asia in the 18th, 19th and first half of the 20th century. UT, along with AM and TCM is considered a prominent traditional medicine system by the World Health Organization (2002). UT as does AM and TCM, recognizes that the human being must be considered a whole composed of entities such as the heart, body, soul or spirit and intellect (Sheehan and Hussain, 2002). Ailments in one part (e.g., brain, mind or intellect) can be caused by diseased states in another part (e.g., heart or emotional state). Therefore, the whole person must be considered when treating a given disorder. Currently, UT has not yet revived to its pre-colonial standard, resulting in few currently available modern empirical research UT publications, though UT studies are steadily increasing (Sheehan and Hussain, 2002). However, there has survived a vast body of historical experiential and evidential literature published by hakims spanning the entire Muslim world, that elaborate on the clinical effectiveness of Islamic SMPs such as dhikr (Zahir, 2022). Encouragingly these are beginning to be translated and published in the English language (Sheehan and Hussain, 2002; Jabin, 2011), with a concomitant movement to revive the hakim tradition.

Complimentary to hakims, another prominent cohort that prescribe dhikr are the ‘murshids’ or ‘murads’. These are spiritual guides, often from the Sufi tradition who act to maintain general wellbeing among people. They do this primarily by looking after the spiritual state of their adherents (and any who come to them seeking guidance), which is understood to effect optimum mental and physical wellbeing. In fact, Sufi healers in many non-Muslim majority countries are sought after by people of other faiths (Singh and Sharan, 2022). However, much like the traditional hakims, qualified and accredited murads are currently a rare breed. Nevertheless, they are also witnessing a post-colonial revival in the Muslim world, and in non-Muslim countries as well (Nurbakhsh, 1978; Ridgeon, 2015; Mitha, 2019). This tradition also richly evidences the effectiveness of dhikr on health and wellbeing (Chishti, 1985). Though again, relevant literature is seldom found in the English language, and there is currently a dearth of rigorous empirical studies conducted using modern scientific methods.

### Research evidence for effectiveness of Islamic SMPs as a psychotherapy for mental illnesses

Recent studies published in the English language show that Islamic dhikr SMPs are powerful reducers of stress and anxiety. A growing body of empirical data underscores the efficacy of simply listening to Quranic recitation as a reducer for anxiety and stress parameters regardless of the faith or religiosity of the participant. A number of relevant publications, including scoping and systematic reviews with rigorous inclusion and exclusion criteria as well as primary research studies are cited for the interested reader (Babamohamadi et al., 2015; Frih et al., 2017; Nasiri et al., 2017; Ghiasi and Keramat, 2018; Yadak et al., 2019; Che Wan Mohd Rozali et al., 2022; Zarea Gavgani et al., 2022; Zulkifli et al., 2022; Moulaei et al., 2023). To summarize their conclusions; remarkably, almost all studies show that simply listening to Quranic recitation (regardless of which portion of the Quran) is able to reduce stress and anxiety biomarkers. The same effect was found in test populations as diverse as patients on dialysis, patients in intensive care units, prisoners, athletes, students taking examinations and pregnant ladies. This finding is compelling because listening to Quranic recitation as an SMP is amenable to easy implementation as a universal CAM intervention. In most cases it simply involves giving the patient a set of earphones and playing them a section of a recorded tape of Quran recitation, even when they may be engaged in other activities.

Yet another Islamic SMP for which there is a growing body of English language publications, is that documenting the competence of dhikr regimens as treatment for various forms of substance abuse. Studies published are predominantly from Indonesia and Malaysia, where there still exist centers of Islamic Sufi healing, which may be equated to modern day therapy centers or psychotherapy clinics. Inpatients are treated with tailored dhikr regimens, which are reported to be more amenable to drug addicts, as they are less intimidating than other forms of SMPs. Dhikr regimens used include, salat, breathing techniques (focus on Divine compassion with the breath), contemplation (‘fikr’)-, self-awareness (‘muraqaba’)-, and self-accountability (‘muhasaba’)- dhikr meditations, recitations of the sacred names of the Divine (‘asma ul husna dhikr’), and ritualized bathing forms of dhikr (‘ghusl’ and ‘wudu’) among others. Overall, studies show positive results, though rigorous empirical molecular data is lacking. Reduced substance dependencies, increased self-awareness and self-control, as well as increased endorphin levels (a biomarker associated with reduced stress and anxiety) were some of the positive empirical impacts reported (Gilman, 1947; Istiqomah, 2013; Mulyati and Nihayah, 2020; Nafisa et al., 2017; Saari et al., 2020; Subandi et al., 2022; Sulaiman et al., 2021). It must be noted that there are vastly more studies published in local languages from the Indo-Malay archipelago on this topic, inaccessible to us. Therefore, it may be expected that the efficaciousness of dhikr reported by English language studies, is an underrepresentation of its full potential.

In terms of the universality of dhikr as a treatment, the fact that both Malaysia and Indonesia are Muslim majority countries likely equates to the vast majority of their participants being of Muslim faith. However, other centers of Sufi psychotherapeutic healing from non majority-Muslim countries such as India for example, note that dhikr therapies are successfully administered for non-Muslim clients even to treat severe psychological conditions (Singh and Sharan, 2022).

An interesting study conducted in the USA also evidences the universal applicability of dhikr. Bahadorani et al. subjected participants, all students at Berkely University (not specific to any religious identity) as a test cohort to an Islamic SMP termed ‘Tamarkoz dhikr’ which involves heart-focussed and movement-based meditations, visualizations, deep breathing, and mind relaxation techniques, and a control cohort, to the University’s standard stress management program (Bahadorani et al., 2021). Remarkably, the Tamarkoz dhikr group reported lower stress levels and higher positive emotions compared to the control cohort. These findings though preliminary, evidence how Islamic dhikr SMPs can be administered universally as a mental health and wellness practice in the modern world.

## Discussion

The ability of spiritual practices to affect wellbeing is indisputable. Epigenetics is proving to be a versatile mechanism that explains some of the modalities by which spiritual and meditative practices can affect gene function, and thus health and well-being. Furthermore, the impact of religion, faith and spirituality on biology has spawned fascinating new research. Neuroimaging has revealed distinct changes in brain regions during practices such as prayer and meditation. Interestingly, these studies have also identified differences in brain activity between secular forms of meditation and those rooted in religious traditions (Al-Nuaimi et al., 2020). They have reinforced the acceptance of SMPs as CAM therapies by explaining molecular modes of action.

Over the past millennium and a half, SMPs rooted in the Islamic spiritual axis have been used by people from across the globe for healing, and maintenance of a happy and healthy life. Sadly, the colonial era that ended the Islamic golden age, witnessed a great decimation of advanced knowledge systems in the Muslim world, and along with it, familiarity with Islamic SMPs diminished. This is a great loss not just for Muslims but all peoples.

The historical contributions of Islamic SMPs especially in the context of mental illness prevention and treatment is remarkable. From the first recognized hospitals established to treat mentally ill persons in the world (Gorini, 2002; Kaf Al-Ghazal et al., 2007; Alkadhat, 2020), to advanced understanding of how environment plays an impact in treatment, pre-colonial Muslim civilization was at the vanguard of explaining mental illness and its treatment. Muslim institutions established to treat mental patients even recognized that music could be an effective treatment (Gurbuz-Dogan et al., 2021), especially noting the prophylactic and therapeutic power of spiritual chants (Istiqomah, 2013; Saniotis, 2018).

These aspects of Islamic SMPs which bring about psychological resilience are in great need of study and revival, as a possible competent psychotherapy for today. They must be examined, and tested thoroughly in order to translate them as modern therapeutic norms in CAM. While salat and other types of ritualized Islamic worship cannot be translated to secular usage, other spiritual practices, especially Quranic recitation and types of dhikr may be universally implemented. Indeed, listening to Quran recitation is an Islamic SMP that is the most easily accessible, as it does not require any effort unlike most SMPs.

Another aspect of Islamic spiritual practice that cannot be overlooked in this perspective, is the centrality of compassion or ‘Rahma’ in the Muslim tradition. Rahma as a concept is essential to the tradition (Arif, 2021). In fact it is the very foundation upon which all the dhikr modes discussed in this paper [example (Saari et al., 2020)] are built. Compassion is the central premise of modern mindfulness meditative techniques such as mindfulness based self compassion. Therefore while further studies on Islamic SMPs are called for, it would be beneficial to also examine their therapeutic principles from the lens of rahma or compassion as a foundational vehicle for healing (Alharbi and Al Hadid, 2019).

Islamic SMPs have centuries of proven efficacy as prophylactic and treatment regimens in all manner of illnesses, but especially for mental illness. Encouragingly, efforts by modern scientists to translate the rich knowledge and puissant practices of Islamic SMPs into modern psychotherapy is gaining traction – some notable publications focussed on integrating Islamic SMPs as psychotherapies in western clinical psychological practices (especially as pertaining to Muslim patients in critical need of spiritually rooted clinical interventions) are cited for the interested reader (Henry, 2015; Rothman and Coyle, 2018, 2020; Rothman et al., 2018; Carle, 2019; Isgandarova, 2019; Bozorgzadeh and Grasser, 2021). The potential for this area of research to yield universally prescribable healing must be explored in significant ways. We call for such extensive and sophisticated investigation, such that Islamic SMPs may also be translated, as the teachings of Buddhism were to result in ‘mindfulness’. What may be considered the equivalent of mindfulness in the Islamic tradition, termed ‘heartfulness’ is just beginning to be researched (York, 2024). Such effort must be encouraged and broadened in order to fully access the immense yet still hidden potential of Islamic SMPs as a universal health intervention.

## Data Availability

The original contributions presented in the study are included in the article/supplementary material, further inquiries can be directed to the corresponding author.
